# Catalpol-Induced AMPK Activation Alleviates Cisplatin-Induced Nephrotoxicity through the Mitochondrial-Dependent Pathway without Compromising Its Anticancer Properties

**DOI:** 10.1155/2021/7467156

**Published:** 2021-01-15

**Authors:** Jiangnan Zhang, Tingting Zhao, Changyuan Wang, Qiang Meng, Xiaokui Huo, Chong Wang, Pengyuan Sun, Huijun Sun, Xiaodong Ma, Jingjing Wu, Kexin Liu

**Affiliations:** ^1^Department of Clinical Pharmacology, College of Pharmacy, Dalian Medical University, Dalian, Liaoning, China; ^2^Provincial Key Laboratory for Pharmacokinetics and Transport, Liaoning Dalian Medical University, Dalian, Liaoning, China

## Abstract

Nephrotoxicity is a common complication of cisplatin chemotherapy and, thus, limits the clinical application of cisplatin. In this work, the effects of catalpol (CAT), a bioactive ingredient extracted from Rehmannia glutinosa, on cisplatin-induced nephrotoxicity and antitumor efficacy were comprehensively investigated. Specifically, the protective effect of CAT on cisplatin-induced injury was explored in mice and HK-2 cells. *In vivo*, CAT administration strikingly suppressed cisplatin-induced renal dysfunction, morphology damage, apoptosis, and inflammation. *In vitro*, CAT induced activation of adenosine 5′-monophosphate- (AMP-) activated protein kinase (AMPK), improved mitochondrial function, and decreased generation of cellular reactive oxygen species (ROS), leading to a reduction in inflammation and apoptosis, which ultimately protected from cisplatin-induced injury. However, the beneficial effects of CAT were mostly blocked by coincubation with compound C. Furthermore, molecular docking results indicated that CAT had a higher affinity for AMPK than other AMPK activators such as danthron, phenformin, and metformin. Importantly, CAT possessed the ability to reverse drug resistance without compromising the antitumor properties of cisplatin. These findings suggest that CAT exerts positive effects against cisplatin-induced renal injury through reversing drug resistance via the mitochondrial-dependent pathway without affecting the anticancer activity of cisplatin.

## 1. Introduction

Cisplatin, an effective and reliable DNA toxin, is widely used against many kinds of solid tumors, including esophageal [1], lung [2], gastric [3], bladder, and liver cancer [4]. However, clinically, cisplatin use is limited by its adverse effects. Approximately 25-40% of clinical patients experience nephropathy following a single injection of cisplatin [5]. Thus, it is critical that new approaches are developed to prevent patients treated with cisplatin from developing kidney toxicity.

Cisplatin-induced acute kidney injury (AKI) is characterized by dilation of renal tubules, extensive necrosis, and exfoliation of epithelial cells; these contribute to a loss of kidney functions, including severe decreases in glomerular filtration and creatinine (CRE) clearance and increases in serum CRE and blood urea nitrogen (BUN) [6]. In addition, an obvious increase in the kidney index (kidney weight/body weight) has been observed in mice treated with cisplatin [7]. Further, the upregulation of kidney injury molecule-1 (KIM-1), a nephrotoxic biomarker, has been observed in cisplatin-treated mice, indicating severe kidney injury [8]. The mechanisms underlying cisplatin-induced AKI involves DNA damage and mitochondrial dysfunction followed by oxidative stress, apoptosis, and necrosis [9]. Numerous studies have demonstrated that cisplatin infiltrates into cells causing DNA damage and leading to massive production of reactive oxygen species (ROS) [10]. ROS accumulation impairs mitochondrial function, producing a reduction in ATP levels and mitochondrial membrane potential together with inhibition of oxidative phosphorylation [11]. This, in turn, triggers inflammation [12] by elevating inflammatory cytokines, such as tumor necrosis factor-*α* (TNF-*α*), interleukin-6 (IL-6), and interleukin1-*β* (IL1-*β*), and results in upregulation of apoptosis via modulation of the expression of Bax and Bcl-2 [13]. In addition, mitochondrial dysfunction reduces mitophagy, aggravating kidney damage [14].

Catalpol (CAT), an active ingredient of the traditional Chinese herbal medicine Rehmannia glutinosa, possesses a variety of pharmacological activities including anti-inflammatory, antiapoptotic, antitumor, and antidiabetes effects [15]. Recent evidence indicates that CAT exerts cardioprotective effects via the mitochondrial-dependent pathway, which is involved in the regulation of Bcl-2 and Bax expression [16]. Additionally, CAT is reported to reduce mitochondrial damage and attenuate atherosclerosis by activating the peroxisome proliferator-activated receptor-*γ* coactivator-1/telomerase reverse transcriptase (PGC-1/TERT) pathway [17]. CAT is also reported to decrease the expression of fission protein 1 and dynamin-related protein 1, increase the expression of mitofusin 1, and improve mitochondrial function, contributing to hepatic protection in diabetic mice [18]. Interestingly, the above effects of CAT are related to mitochondrial function. Our previous study confirmed a kidney-protective effect of CAT via activation of the SIRT1 signaling pathway [19]. However, the effect of CAT on cisplatin-induced AKI via the mitochondrial pathway remains unknown.

In this study, we explored the effects of CAT on cisplatin-induced AKI in mice and revealed the underlying molecular mechanisms. We also confirmed the role of mitochondria in cisplatin-induced nephrotoxicity.

## 2. Material and Methods

### 2.1. Reagents

CAT (98%) was obtained from Nanjing Jingzhu Biotech Co., Ltd (Nanjing, China). Cisplatin was purchased from Solarbio Life Science (Beijing, China). The adenosine 5′-monophosphate- (AMP-) activated protein kinase (AMPK) inhibitor, Compound C, and the AMPK activator, AICAR (5-aminoimidazole-4-carboxamide ribonucleotide), were purchased from Selleck Chemicals (USA). Cell viability was assessed using a Cell Counting Kit-8 (CCK-8) provided by Biomarker (USA). The human proximal tubule epithelial cell line (HK-2) was purchased from the Beina Technology Co., Ltd. A549-sensitive (A549), A549-resistant paclitaxel (A549/PTX), and A549-resistant cisplatin (A549/DDP) cells were obtained from KenGen BioTechnology Co., Ltd (Nanjing, China). All other materials are commercially available unless otherwise stated.

### 2.2. Animal Experiments

A total of 36 adult male Kunming mice (6-8 weeks; 20-25 g) were obtained from the Laboratory Animal Center of Dalian Medical University (Dalian, China; permit number SYXK 2018-0007). All animal studies were conducted in accordance with the Guidelines for the Care and Use of Laboratory Animals of the National Institutes of Health. All animal experiments satisfied the standard for reduction, refinement, and replacement (the 3Rs). All mice had free access to food and water and were fed adaptively for seven days. The animals were randomly divided into six groups, *n* = 6 per group: group A (control group), group B (CAT, 80 mg/kg), group C (cisplatin, 25 mg/kg), group D (cisplatin, 25 mg/kg + CAT, 40 mg/kg), group E (cisplatin, 25 mg/kg + CAT, 80 mg/kg), and group F (cisplatin, 25 mg/kg + AICAR, 500 mg/kg). Cisplatin was given by a single intraperitoneal injection on the first day of the experiment for groups C, D, E, and F [20]. Then, CAT or AICAR were given by intragastric administration, daily for three days (CAT for groups B, C, D, and E; AICAR for group F). The control group was given an equal volume of normal saline. After the three days of cisplatin injection, mice were anesthetized with pentobarbital sodium (40 mg/kg) and then sacrificed by cervical dislocation. Blood samples were obtained from the abdominal aorta after the mice were anesthetized with sodium pentobarbital. The weights of the kidneys and body were recorded. Serum and kidney tissue were collected for further measurements.

### 2.3. Biochemical Analysis

Serum CRE and BUN concentrations, as well as superoxide dismutase (SOD), malondialdehyde (MDA), and glutathione (GSH) levels in the kidney tissue, were determined with commercial assay kits purchased from Nanjing Jiancheng Institute of Biotechnology (Nanjing, China). All experimental protocols were performed according to the manufacturers' protocols. Absorbance values for these measurements were detected by a microplate reader (Tecan, Austria). The SOD, MDA, and GSH values of the kidney were normalized by the tissue protein concentrations.

### 2.4. Kidney Histopathological Assay

The left kidney cortex was cut for histopathology and was immersed in 10% neutral formalin overnight. Then, it was embedded in paraffin and sliced into 5 *μ*m tissue slices. The sections were stained with a hematoxylin-eosin (HE) staining kit (Nanjing Jiancheng Institute of Biotechnology, Nanjing, China) and TUNEL staining kit (Promega, USA) for general histology and apoptosis examination, respectively. Sections were detected by microscopy (Olympus OIS IX81, Japan), and images were captured in random fields.

### 2.5. Cell Culture and Experimental Design

HK-2 cells were cultured in DMEM/F12 (1 : 1) containing 10% (*v*/*v*) fetal bovine serum (FBS). A549, A549/PTX, and A549/DDP were cultured in 1640 (1 : 1) containing 10% (*v*/*v*) FBS. All cells are cultured at 37°C under 95% humidity and 5% CO_2_. After two days of culture, HK-2 cells were seeded into the plate and grown in a complete medium. When the cells had grown to approximately 80% of the plate, CAT was added at corresponding concentrations for 24 h. Then, the cells were incubated for 24 h under coadministration of CAT and cisplatin. A549, A549/PTX, and A549/DDP were seeded into the plate and cultured with a complete medium. Then, the indicated drugs were added, and the cells were incubated for 24 h.

### 2.6. CCK-8 Assay

All cells were seeded into a 96-well plate at a density of 1 × 10^5^ cells/ml. When the cells had grown to approximately 80% of the plate, complete medium was replaced with serum-free medium containing cisplatin, CAT, or the combination of CAT and cisplatin at indicated concentrations for 24 h. Then, 10 *μ*l of CCK-8 solution was added to each well, and the cells were incubated for 2 h at 37°C. The absorbance was assessed at 450 nm with a microplate reader (Tecan, Austria). The IC_50_ value was calculated with Prism GraphPad.

### 2.7. ELISA Kit

HK-2 cells were cultured in a six-well plate. After treatment with the indicated drugs, the cells were collected with lysate buffer for assay. Mouse plasma was centrifuged, and the supernatant was used for the assay. The concentrations of TNF-*α*, IL-6, IL1-*β*, and KIM-1 were determined with the indicated ELISA kit (Proteintech Group, Wuhan, China), according to the manufacturers' protocols. The values of cell TNF-*α*, IL-6, and IL1-*β* were normalized by the cell protein concentrations.

### 2.8. Western Blot

10 mg of kidney cortex tissue was used for western blot assay. The tissue was diluted with lysis buffer (1 : 9, mg/*μ*l) and incubated for 20 min at 4°C. The HK-2 cells were seeded into a six-well plate and were treated with specific drugs. Then, the cells were collected with 200 *μ*l lysate buffer and incubated for 20 min at 4°C. All samples were centrifuged (12000 rpm, 15 min), and the supernatant was collected. The protein concentration was determined by a BCA kit (Solarbio Life Science, Beijing, China), according to the manufacturer's instructions. The western blotting assay was conducted with 40 *μ*g total protein. Primary antibodies against AMPK (NO.5831; 1 : 1000), P-AMPK (NO.2535; 1 : 1000), and Bax (NO.2772; 1 : 1000) were obtained from Cell Signaling Technology (Beverly, USA). Primary antibodies against *β*-actin (NO.66009-1-Ig; 1 : 1000), Bcl-2 (NO.12789-1-AP; 1 : 1000), and LC-3 (NO.14600-1-AP; 1 : 1000), as well as the HRP-conjugated secondary antibodies (Anti-Rabbit NO. SA00001-2; Anti-Mouse NO. SA00001-1; 1 : 3000), were purchased from Proteintech Group (Wuhan, China). The protein bands were detected with a Tanon-5200 Imaging system, and the densities of the bands were determined by the ImageJ 1.8 software. *β*-Actin was chosen for normalization of relative protein expression due to its high and constant expression.

### 2.9. Intracellular ROS Assay

Levels of intracellular ROS were measured with a ROS Assay Kit (Beyotime Biotechnology, China), according to the manufacturer's protocol. Briefly, HK-2 cells were treated with the indicated drugs and incubated with serum-free medium containing DCFH-DA (10 *μ*M) for 20 min at 37°C. The fluorescence intensity was assessed using a BD FACS Calibur system (Becton, Dickinson and Company, USA) with an excitation wavelength of 488 nm and an emission wavelength of 525 nm.

### 2.10. Immunofluorescence

LysoTracker and MitoTracker were obtained from Cell Signaling Technology (Beverly, USA) to assess the level of mitophagy. HK-2 cells were cultured in a six-well plate and treated with specific drugs. After incubation according to the manufacturer's protocol, the plate was detected by microscopy, and images were captured in random fields. Red fluorescence represents mitochondria, and green fluorescence represents lysosomes.

### 2.11. Cell Apoptosis Assay

An annexin V/7-AAD Double Staining Kit (Thermo Fisher Scientific, USA) was employed to explore cell apoptosis. After treatment with indicated reagents, cells were collected following trypsinization and centrifugation. Annexin V-FITC (10 *μ*l) was added, and the cells were incubated for 20 min at room temperature in the dark. Next, 7-AAD (5 *μ*l) solution was added. After coincubation for 10 min, the fluorescence values were detected by flow cytometry. Moreover, a TUNEL Staining Kit was used to measure the apoptotic rate; green fluorescence represents apoptotic cells, and blue fluorescence represents total cells. The apoptotic rate = apoptotic cells/total cells.

### 2.12. Measurement of Intracellular ATP

HK-2 cells were cultured in a six-well plate and then treated with specific drugs. ATP content was measured using an ATP Assay Kit (Beyotime Biotechnology, China). The procedure was performed according to the manufacturer's protocol. The ATP value was normalized by the cell protein concentrations.

### 2.13. JC-1 Staining

The mitochondrial membrane potential was evaluated using a JC-1 Kit (Solarbio Life Science, Beijing, China), according to the manufacturer's instructions. Briefly, after treatment with reagents, HK-2 cells were incubated with serum-free medium containing JC-1 for 20 min at 37°C in the dark. Then, the plate was detected by a fluorescence microscope, and images were captured in random fields.

### 2.14. Molecular Docking

The Sybyl/Surflex module was employed to investigate the potential interactions between CAT and AMPK. The AMPK protein crystal structure (PDB ID: 5UFU) was downloaded from the protein data bank (http://www.rcsb.org/). The AMPK activators, including danthron, metformin, and phenformin, were obtained from http://zinc15.docking.org. All docking simulations were performed with default parameters, and binding affinities were estimated by total scores. The docking results were further visualized with a 2D molecular descriptor using the Discovery Studio Visualizer software, version 16.1.0.

### 2.15. Statistical Analysis

In general, the data are presented as mean ± standard deviation (S.D.) and were analyzed using the Prism program (version 5.0.1, GraphPad, San Diego, CA). Statistically significant differences were determined by one-way ANOVA followed by Tukey's post hoc tests or unpaired *t*-tests. Statistically significant differences were indicated by *P* < 0.05.

## 3. Results

### 3.1. Effects of CAT on Cisplatin-Induced AKI in Mice

**Figure 1:**
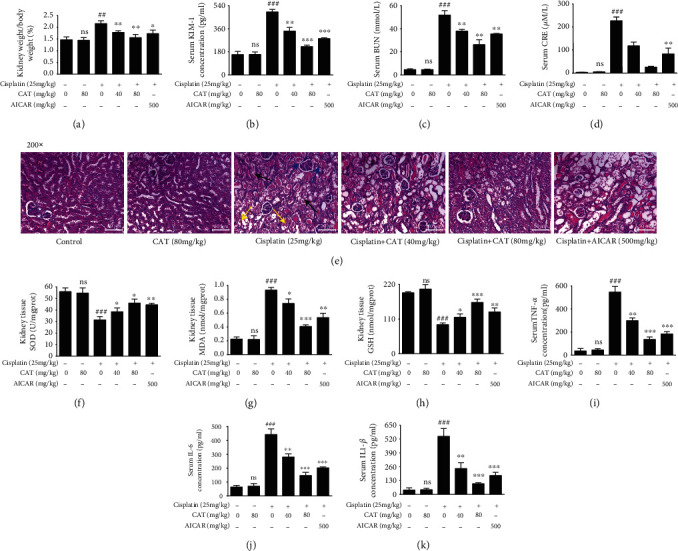
CAT attenuated cisplatin-induced nephrotoxicity (*in vivo*). Changes in kidney weight/body weight (a), serum KIM-1 (b), serum BUN (c), serum CRE (d), TUNEL staining (e), kidney tissue SOD (f), MDA (g), GSH (h), serum TNF-*α* (i), serum IL-6 (j), and serum IL1-*β* (k) after coadministration of CAT. Data were analyzed by one-way ANOVA and unpaired *t*-tests and are presented as mean ± SD. ^##^*P* < 0.01 and ^###^*P* < 0.01*vs.* the control group; ^∗^*P* < 0.05, ^∗∗^*P* < 0.01, and ^∗∗∗^*P* < 0.001*vs.* the model group; ns: not significant (*P* > 0.05*vs.* the control group) (*n* = 5).

To investigate the protective effect of CAT on renal injury induced by cisplatin, kidney weight, body weight, and serum biochemical factors were examined in mice. As shown in Figure 1, the kidney-to-body weight ratio was increased in the cisplatin group, and CAT prevented, but did not normalize, the cisplatin-induced increase (Figure 1(a)). Further, the serum levels of KIM-1, BUN, and CRE were upregulated by cisplatin but were markedly reduced by CAT in a dose-dependent manner (Figures 1(b)–1(d)). Moreover, the HE staining results indicated that in the cisplatin group, tubular dilatation (yellow arrow), loss of epithelial cells (black arrow), and extensive necrosis (blue arrow) occurred in the tubules. In contrast, CAT treatment dose-dependently alleviated tubular injury (Figure 1(e)). More importantly, CAT alone did not affect the kidneys of mice and CAT (80 mg/kg) showed better reversal effects in all examinations as compared to AICAR. These data indicate that CAT may improve renal function in mice exposed to cisplatin.

### 3.2. CAT Attenuated Oxidative Stress Induced by Cisplatin in Mice

Assays of kidney tissue SOD, MDA, GSH, and serum cytokines were conducted to evaluate oxidative stress in the mice. The data indicated that cisplatin induced an increase in MDA (Figure 1(g)), TNF-*α* (Figure 1(i)), IL-6 (Figure 1(j)), and IL1-*β* (Figure 1(k)) and a decrease in SOD (Figure 1(f)) and GSH (Figure 1(h)). CAT treatment significantly reversed this trend in a dose-dependent manner, including increasing SOD and GSH as well as reducing MDA, TNF-*α*, IL-6, and IL1-*β*. Importantly, the CAT group exhibited no significant difference compared with the control group, and CAT (80 mg/kg) produced better reversal in all assays as compared to AICAR. Taken together, these results indicate that CAT produces a reduction in inflammatory cytokines by modulating oxidative stress.

### 3.3. CAT Activated AMPK, Reduced Apoptosis, and Increased Autophagy *In Vivo*

**Figure 2:**
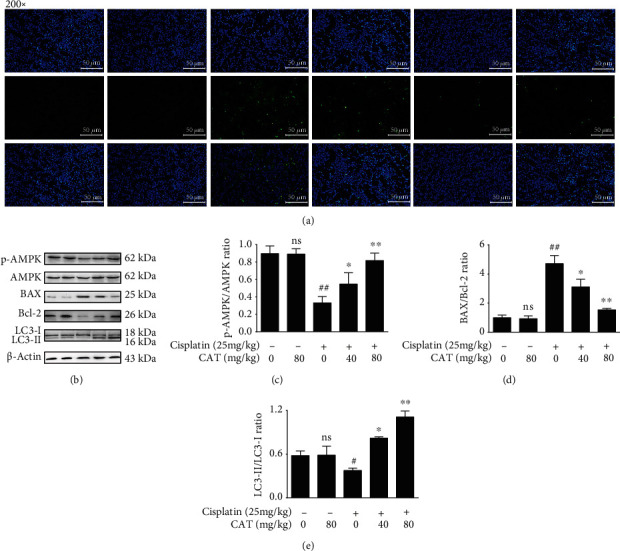
CAT inhibited apoptosis and activated autophagy *in vivo*. Changes in kidney apoptosis (a), AMPK expression (b), BAX/Bcl-2 ratio (c), and LC-3 expression (d) after coadministration of CAT. Data were analyzed by one-way ANOVA with unpaired *t*-tests, and are presented as mean ± SD. ^#^*P* < 0.01 and ^##^*P* < 0.01*vs.* the control group; ^∗^*P* < 0.05 and ^∗∗^*P* < 0.01*vs.* the model group; ns: not significant (*P* > 0.05*vs.* the control group) (*n* = 5).

To examine the apoptotic response of AKI mice, TUNEL staining was performed. Few TUNEL-positive cells were observed in the control and CAT group. However, cisplatin induced an increase in the number of TUNEL-positive cells, and this was significantly blocked by CAT treatment in a dose-dependent manner. Moreover, the AICAR group showed more TUNEL-positive cells than the cisplatin+CAT (80 mg/kg) group (Figure 2(a)). Consistently, the regulation of apoptosis-associated proteins Bcl-2 and Bax was strikingly reversed by coadministration of CAT (Figure 2(c)). Additionally, cisplatin reduced the relative expression of AMPK and LC-3, while the CAT combination upregulated AMPK and LC-3 protein expression (Figures 2(b) and 2(d)). These findings suggest that activation of AMPK by CAT might regulate apoptosis and autophagy activity *in vivo*.

### 3.4. CAT Inhibited Cisplatin-Induced Apoptosis in HK-2

**Figure 3:**
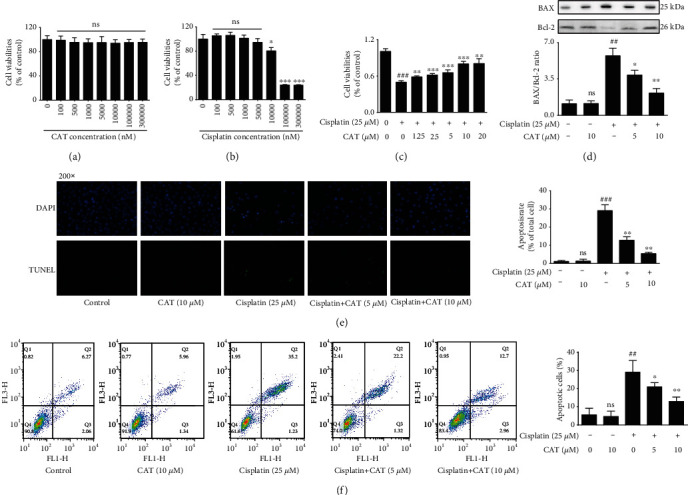
CAT attenuated cisplatin-induced damage by inhibiting apoptosis *in vitro*. Cell viability after treatment with various concentrations of CAT (a), cisplatin (b), and coadministration of CAT and cisplatin (c). Changes in the BAX/Bcl-2 ratio (d), TUNEL staining (e), and flow cytometry (f) after cotreatment of CAT and cisplatin. Data were analyzed by one-way ANOVA and unpaired *t*-tests and are presented as mean ± SD. ^##^*P* < 0.01 and ^###^*P* < 0.001*vs.* the control group; ^∗^*P* < 0.05, ^∗∗^*P* < 0.01, and ^∗∗∗^*P* < 0.001*vs.* the cisplatin group; ns: not significant (*P* > 0.05 vs. the control group) (*n* = 5).

A cell cytotoxicity assay was conducted using a CCK-8 kit to investigate cell viability under CAT, cisplatin, and both. After treatment with CAT at various concentrations from 100 nM to 100 *μ*M for 24 h, we observed no toxic effects on HK-2 cells, even at a concentration of 100 *μ*M (Figure 3(a)), while cell viability gradually declined when HK-2 cells were exposed to increasing concentrations of cisplatin from 10 *μ*M to 300 *μ*M, and the IC_50_ value of cisplatin was calculated to be 25 *μ*M (Figure 3(b)). Remarkably, CAT produced striking inhibition of cell cytotoxicity with increasing concentration (Figure 3(c)). In parallel to reduced apoptotic cells, the increase in Bax expression was markedly blocked, and the reduction in Bcl-2 expression was distinctly attenuated by cotreatment with CAT in cisplatin-exposed HK-2 cells (Figure 3(d)). Furthermore, TUNEL staining and flow cytometry indicated that cisplatin-induced apoptosis was inhibited in a concentration-dependent manner when combined with CAT treatment (Figures 3(e) and 3(f)). These results suggest that CAT inhibits cisplatin-induced apoptosis in HK-2 cells.

### 3.5. CAT Improved Mitochondrial Function in HK-2 Cells

**Figure 4:**
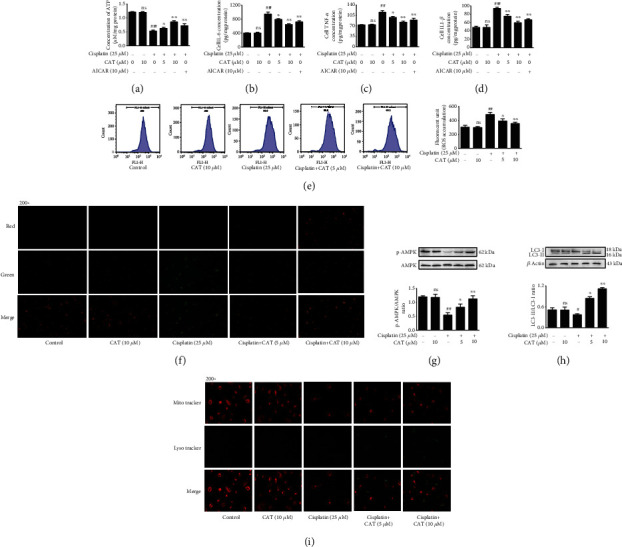
CAT activated AMPK and alleviated cisplatin-induced mitochondrial dysfunction by inhibiting ROS production *in vitro*. Changes in cellular ATP (a), cell IL-6 (b), cell TNF-*α* (c), cell IL1-*β* (d), cellular ROS (e), mitochondrial membrane potential (f), AMPK expression (g), LC-3 expression (h), and mitophagy activity (i) after cotreatment of CAT and cisplatin. Data were analyzed by one-way ANOVA and unpaired *t*-tests and are presented as mean ± SD. ^##^*P* < 0.01 and ^###^*P* < 0.001 vs. the control group; ^∗^*P* < 0.05 and ^∗∗^*P* < 0.01*vs*. the cisplatin group; ns: not significant (*P* > 0.05*vs.* the control group) (*n* = 5).

To investigate mitochondrial function, we first assessed the ATP content of HK-2 cells. As shown in Figure 4, cisplatin decreased the intracellular ATP level. Consistently, cisplatin induced enhancement of TNF-*α*, IL-6, and IL1-*β* concentrations, and the effects of cisplatin were mostly abolished with CAT or AICAR treatment, ultimately leading to normalization of ATP and inflammatory cytokine levels. Importantly, at a concentration of 10 *μ*M, CAT exhibited an equal or better effect than AICAR (positive control drug) (Figures 4(a)–4(d)). Similarly, the flow cytometry analysis showed a massive production of intracellular ROS in the cisplatin group. After coincubation with cisplatin and CAT, cellular ROS accumulation was eliminated in a concentration-dependent manner (Figure 4(e)). Moreover, the control and the CAT groups showed light red fluorescence intensity and relative weak green fluorescence intensity, while an increase in green fluorescence intensity and a decrease in red fluorescence intensity occurred when the cells were treated with cisplatin for 24 h. In contrast, red fluorescence gradually brightened, while green fluorescence relatively weakened under cotreatment with CAT and cisplatin (Figure 4(f)). These results indicate that the improvement in mitochondrial function induced by CAT reduces inflammation in HK-2 cells.

### 3.6. CAT Activated AMPK and Increased Mitophagy in HK-2 Cells

To investigate changes in autophagy upon treatment with cisplatin, HK-2 cells were subjected to western blot assay followed by protein band density analysis. Cisplatin treatment contributed to hypophosphorylation or inhibition of AMPK, resulting in inhibition of expression of the autophagy-related protein LC-3. CAT preincubation blocked the effect of cisplatin and restored phosphorylation or activation of AMPK in a concentration-dependent manner, accompanied by reinstatement of LC3 expression (Figures 4(g) and 4(h)). Immunofluorescence analysis was performed to further evaluate the level of mitophagy. In parallel to the activation of AMPK and reinstatement of LC3 expression, CAT treatment normalized the fluorescence intensity of mitoTracker and lysotracker in a concentration-dependent manner (Figure 4(i)). These findings suggest that phosphorylation of AMPK by CAT might activate mitophagy.

### 3.7. AMPK Activation Is Required for the Effect of CAT in HK-2 Cells

**Figure 5:**
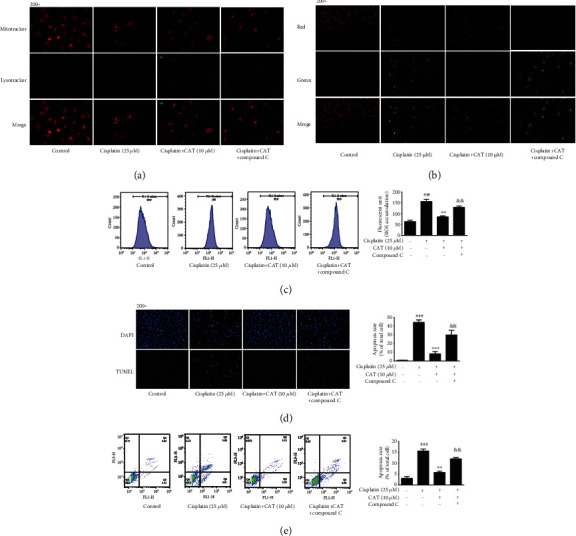
AMPK is involved in the effect of CAT on mitophagy, ROS, and apoptosis. HK-2 cells were treated with CAT, CAT and Compound C, or free-fetal medium. Then, the cells were treated with the indicated drugs or complete medium. The changes in mitochondrial membrane potential (a), mitophagy activity (b), and ROS accumulation (c) are shown. Furthermore, the changes in apoptosis were assessed by TUNEL staining (d) and flow cytometry (e). Data were analyzed by one-way ANOVA and unpaired *t*-tests and are presented as mean ± SD. ^###^*P* < 0.001*vs.* the control group. ^∗∗^*P* < 0.01*vs*. the cisplatin group. ^&&^*P* < 0.01*vs.* the cisplatin+CAT group (*n* = 5).

To determine whether AMPK was responsible for the effect of CAT on cisplatin-induced damage in HK-2 cells, cultured cells were preincubated with CAT at different concentrations with or without the AMPK-specific inhibitor Compound C and then were treated with cisplatin for 24 h. Notably, CAT treatment strikingly restored autophagic activity and mitochondrial membrane potential changes. In contrast, CAT incubation significantly suppressed the production of cellular ROS caused by cisplatin, while these effects of CAT were partly abolished following pretreatment of HK-2 cells with Compound C, resulting in a reduction in mitophagy activity and mitochondrial membrane potential as well as an increase in ROS production (Figures 5(a)–5(c)). Furthermore, cell apoptosis was detected, and the flow cytometry assay revealed an increased apoptotic rate after treatment with Compound C, as compared with the cisplatin+CAT group (Figure 5(d)). Consistently, TUNEL staining indicated that CAT reversed the increase in the apoptotic ratio and reduction in total cell number induced by cisplatin, while the beneficial effect was prominently blocked by coincubation with Compound C (Figure 5(e)).

### 3.8. Interaction between CAT and AMPK Evaluated by Docking Studies

**Figure 6:**
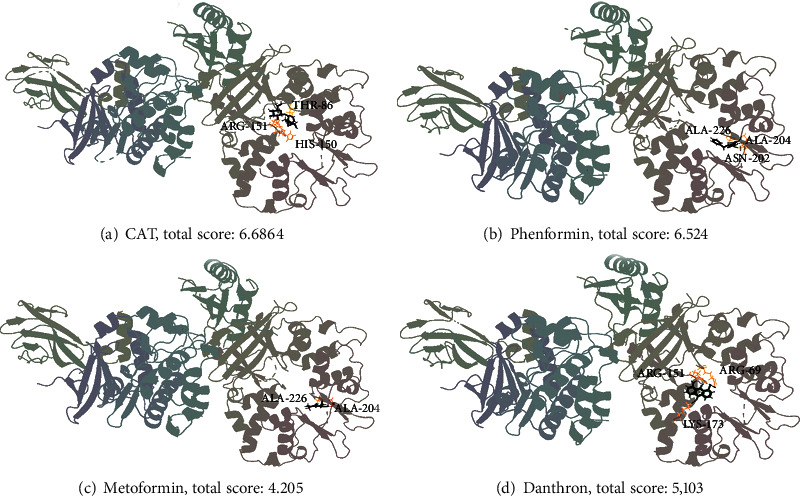
Molecule docking simulation of compounds with 5UFU. Catalpol (a), phenformin (b), metformin (c), and danthron (d) bind to the active site of 5UFU.

To further investigate the molecular interaction between AMPK and CAT, the structure of AMPK (PDB: 5UFU) and classical AMPK activators (including phenformin, metformin, and danthron) was included in molecular simulations. As shown in Figure 6, four H-bond interactions occurred in CAT, phenformin, and danthron with the active pocket of AMPK (Figures 6(a), 6(b), and 6(d)). In contrast, metformin showed three H-bond interactions (Figure 6(c)), resulting in a low total score for metformin. Furthermore, CAT possessed a higher total score than phenformin and danthron. These results suggest that CAT has a stronger affinity for AMPK than other activators, contributing to it being a potential AMPK activator.

### 3.9. CAT Did Not Affect the Anticancer Activity of Cisplatin in A549 and Strengthened the Cytotoxicity of Cisplatin in A549/DDP and A549/PTX

**Figure 7:**
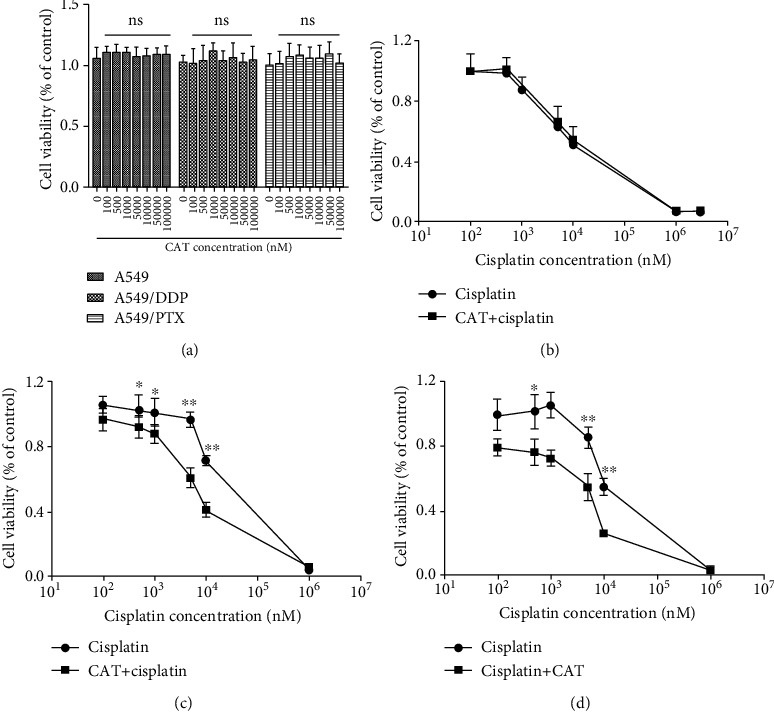
Cytotoxicity of cisplatin and CAT in A549, A549/PTX, and A549/DDP cells. Cells were treated with various concentrations of CAT to examine the cytotoxicity of CAT in three cell lines (a), and the cytotoxicity of cisplatin treatment combined with CAT (10 *μ*M) was assessed in A549 (b), A549/PTX (c), and A549/DDP (d). Data were analyzed by one-way ANOVA and unpaired *t*-tests and are presented as mean ± SD. ns: not significant (*P* > 0.05*vs.* the control group) (*n* = 5).

To explore the effect of CAT on cisplatin anticancer properties, cytotoxicity assays were performed in A549, A549/DDP, and A549/PTX cells. CAT had no toxic effects on the three cell types, even at a high concentration of 100 *μ*M (Figure 7(a)), and the IC_50_ values in A549 were not significantly altered between cisplatin treatment and coadministration of CAT with cisplatin (Figure 7(b), Table 1). Further, cotreatment with CAT and cisplatin enhanced the cytotoxicity of cisplatin to A549/DDP and A549/PTX cells (Figures 7(c) and 7(d)), supported by a decrease in the IC_50_ values and resistance index. The reversal indices were 2.8 and 4.15 in A549/PTX and A549/DDP cells, respectively, when cotreated with CAT (Table 1). These results demonstrate that CAT does not interfere with the anticancer activity of cisplatin and overcomes drug resistance.

## 4. Discussion

AKI is an abrupt loss of kidney function caused by many clinical factors, including ischemia-reperfusion injury [21], chemotherapy [22], surgery [23], and application of contrast materials for imaging investigations [24]. Recent surveys suggest that a large percentage of hospitalized patients suffer from AKI, which aggravates the economic burden of disease [25]. However, there are no effective therapies for AKI, and prevention is the only choice. Nephropathy, the most common adverse effect of cisplatin, limits its clinical application [26]. To address these sorts of issues, research has turned its focus to compounds extracted from traditional Chinese medicines, and numerous studies indicate that kidney dysfunction can be improved by treatment with the ingredients of Chinese herbs, such as quercetin [27], naringin [28], and formononetin [29]. CAT is one of the active components of Rehmannia glutinosa, and previous studies have confirmed that it has neuroprotective effects due to its powerful antioxidant and anti-inflammatory activities. Moreover, our prior research revealed a nephroprotective effect of CAT on adriamycin-induced injury. Thus, we hypothesized that CAT has the potential to interrupt cisplatin nephropathy. To this end, the present research is the first to verify the effect of CAT on cisplatin-induced AKI. As expected, the kidney weight/body weight rate increased upon injection of cisplatin, and cotreatment with CAT decreased the ratio (Figure 1(a)). Similarly, cisplatin-induced morphological damages to the kidney tubules and kidney dysfunction were significantly alleviated (Figure 1(e)). These *in vivo* data strongly indicate the potential application of CAT for the treatment of cisplatin-induced AKI. Furthermore, CAT restored the reduction in cell viability induced by cisplatin in HK-2 cells (Figure 3(c)). The mechanisms underlying these effects require further exploration.

Recent studies highlight energy substrates as new targets to attenuate AKI and chronic kidney disease (CKD) [30]. AMPK, a cellular energy sensor, is believed to modulate mitochondrial energy production. Activation of AMPK regulates the mitochondrial pathway to ensure ATP production and further sustain kidney energy homeostasis [31]. In this work, cisplatin decreased the expression of AMPK (Figures 2(a) and 4(g)) and impaired mitochondrial function, as indicated by an increase in the mitochondrial membrane potential (Figure 4(f)), resulting in a reduction in ATP content, increase in inflammation and apoptosis, and activation of mitophagy (Figure 4). In contrast, CAT treatment normalized the mitochondrial membrane potential and restored ATP production. Furthermore, the AMPK activating effect of CAT was partly abolished under cotreatment with the AMPK inhibitor Compound C, leading to a reduction in the mitochondrial membrane potential (Figure 5(b)). These data suggest that CAT modulates mitochondrial function via activation of AMPK.

AICAR [32], metformin [33], and neferine [34] are well-known AMPK activators and have been widely used in cisplatin-induced experimental nephrotoxicity studies, with similar results to our study. In the current study, AICAR administration, as a positive control, produced effects similar to CAT administration, including improvement in kidney tubular dilatation, tubular formation, and tubular necrosis induced by cisplatin; however, CAT showed better protective effects (Figure 1). Moreover, it has been confirmed that metformin alleviates cisplatin-induced renal injury by activating AMPK-induced autophagy. Our molecular docking results indicated that CAT has a higher affinity with AMPK than metformin, which may suggest a better therapeutic effect of CAT. More importantly, unlike our study, many of the studies mentioned did not examine the effect of AMPK activators on the anticancer properties of cisplatin, which limits their potential clinical application.

Importantly, this study evaluated whether the combination of CAT and cisplatin affected the antitumor properties of cisplatin. A CCK-8 assay was employed to detect the viability of A549 cells treated with CAT, cisplatin, or both. As shown in Figure 7, CAT produced no toxicity to A549 cells, even at a concentration of 100 *μ*M (Figure 7(a)), and did not affect the cytotoxicity of cisplatin at a concentration of 10 *μ*M, which is the concentration chosen to examine the nephroprotective effects (Figure 7(b)). Furthermore, multidrug resistance (MDR) is a challenging problem in cisplatin chemotherapy, and to date, there are no effective drugs that can be used clinically to overcome MDR. To investigate the MDR reversal effect of CAT, A549/DDP and A549/PTX cells were cotreated with CAT (10 *μ*M) and cisplatin. Notably, coadministration of CAT and cisplatin strikingly enhanced the cytotoxicity of cisplatin to A549/DDP and A549/PTX cells (Figures 7(c) and 7(d)), which was supported by reductions in the IC_50_ values and resistance indices (Table 1). In summary, this study highlights the meaningful and clinically relevant effect of cotreatment with CAT and cisplatin. CAT did not affect the antitumor activity of cisplatin in drug-sensitive cells but strengthened the cytotoxicity of cisplatin in drug-resistant cells, while consistently producing a nephroprotective effect.

It should be noted that our study has several limitations. Cisplatin is a first-line drug for the treatment of many solid tumors [35], but the effect of CAT on the antitumor activity of cisplatin was evaluated only in A549 cells in this study. Thus, follow-up experiments are needed to investigate the role of CAT in a variety of tumor cell lines. This would provide comprehensive evidence that CAT does not affect the antitumor activity of cisplatin, which is important for the clinical application of CAT. Further, we evaluated the effect of CAT on cisplatin antitumor activity at the cellular level. Tumor-bearing nude mice should be employed *in vivo* to assess the effects of CAT on cisplatin-sensitive solid tumors and cisplatin-resistant solid tumors.

## 5. Conclusion

**Figure 8:**
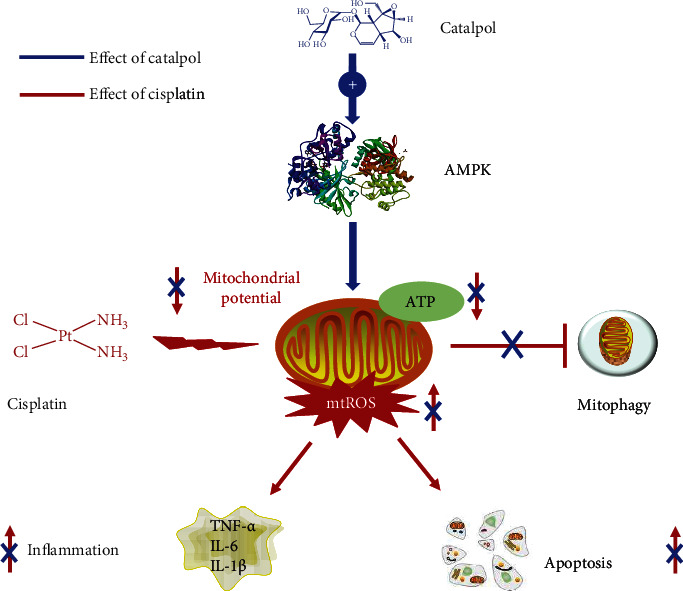
Schematic illustration of the signaling pathway involved in the effect of CAT on cisplatin-induced nephrotoxicity *in vitro*. CAT activated AMPK activity and improved mitochondrial function, decreased cellular ROS generation, and increased ATP production, leading to reduced inflammation and apoptosis as well as activation of mitophagy, which ultimately protected against cisplatin-induced injury.

This study demonstrated the beneficial effects of CAT on cisplatin-induced AKI and provided new insight into the molecular mechanisms of CAT against cisplatin-induced damage. In mice treated with cisplatin, CAT pretreatment markedly improved kidney function, decreased apoptotic cells, and suppressed the levels of inflammatory cytokines. Moreover, CAT attenuated cisplatin-induced injury by normalizing mitochondrial function, leading to enhanced mitophagy activity and reduced ROS accumulation. Furthermore, the protective effect of CAT on cisplatin-induced damage was found to be associated with the mitochondrial-dependent pathway via the activation of AMPK. More importantly, CAT did not affect the antitumor activity of cisplatin in drug-sensitive cells but strengthened the cytotoxicity of cisplatin in drug-resistant cells (Figure 8).

## Figures and Tables

**Table 1:** IC_50_ values and reversal effect of CAT on cisplatin in A549, A549/PTX, and A549/DDP cells.

Cell lines	IC_50_^a^ (*μ*M)		Resistance Index^b^	Reversal Index^c^
	Cisplatin	Cisplatin+CAT (10 *μ*M)		
A549	3.54 ± 0.13	3.76 ± 0.19	—	1
A549/PTX	14.67 ± 0.46	5.25 ± 0.12^##^	4.14	2.8
A549/DDP	28.69 ± 0.81	6.91 ± 0.17^##^	8.10	4.15

^a^IC_50_ values represent the mean ± SD of three independent experiments. ^b^Resistance Index was calculated by dividing the IC_50_ values of the cisplatin treatment in A549/PTX and A549/DDP cells by that of A549 cells. ^c^Reversal Index was calculated by dividing the IC_50_ values of the cisplatin treatment by that of the combined cisplatin with CAT treatment. ^##^*P* < 0.01*vs.* cisplatin group.

## Data Availability

All data generated or analyzed during this study are included in this published article.

## Abbreviations

AMPK: adenosine 5′-monophosphate-activated protein kinase
(AMP): activated protein kinase
BUN: blood urea nitrogen
CAT: catalpol
CRE: creatinine
IL1-*β*: interleukin1-*β*
IL-6: interleukin-6
KIM-1: kidney injury molecule-1
PGC-1/TERT: peroxisome proliferator-activated receptor-*γ* coactivator-1/telomerase reverse transcriptase
ROS: reactive oxygen species
TNF-*α*: tumor necrosis factor-*α*
AKI: acute kidney injury
CCK-8: Cell Counting Kit-8
MDA: malondialdehyde
SOD: superoxide dismutase
GSH: glutathione
HE: hematoxylin-eosin
HK-2: human proximal tubule epithelial cell line
AICAR: 5-aminoimidazole-4-carboxamide ribonucleotide.

## Supplementary Materials

Supplementary Materials The uncropped bolts of all protein blots in Figures.
