# Supplementary material for: Catalpol-Induced AMPK Activation Alleviates Cisplatin-Induced Nephrotoxicity through the Mitochondrial-Dependent Pathway without Compromising Its Anticancer Properties
Source: Oxid Med Cell Longev. 2021 Jan 15;2021:7467156. doi: 10.1155/2021/7467156 (PMC7826214; doi:10.1155/2021/7467156)

supplementary file

**Catalpol-Induced AMPK Activation Alleviates Cisplatin-induced Nephrotoxicity Through Mitochondrial-dependent Pathway Without Compromising Its Anticancer Properties**

Jiangnan Zhang^a, #^, Tingting Zhao^a, #^, Changyuan Wang^a, b, #^, Qiang Meng^a, b^, Xiaokui Huo^a, b^, Chong Wang^a, b^, Pengyuan Sun^a, b^, Huijun Sun^a, b^, Xiaodong Ma^a, b^, Jingjing Wu^a, b, *^, Kexin Liu^a, b, *^


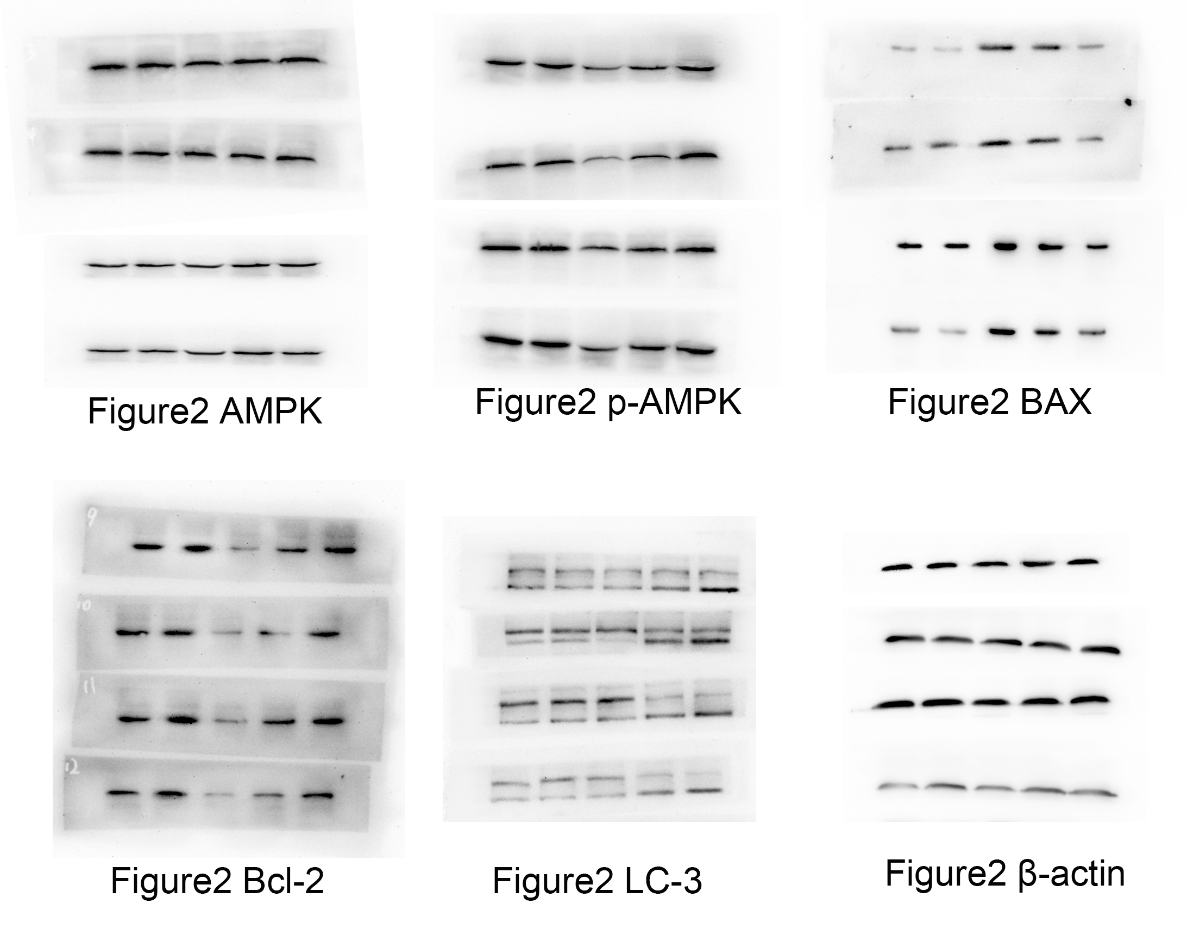


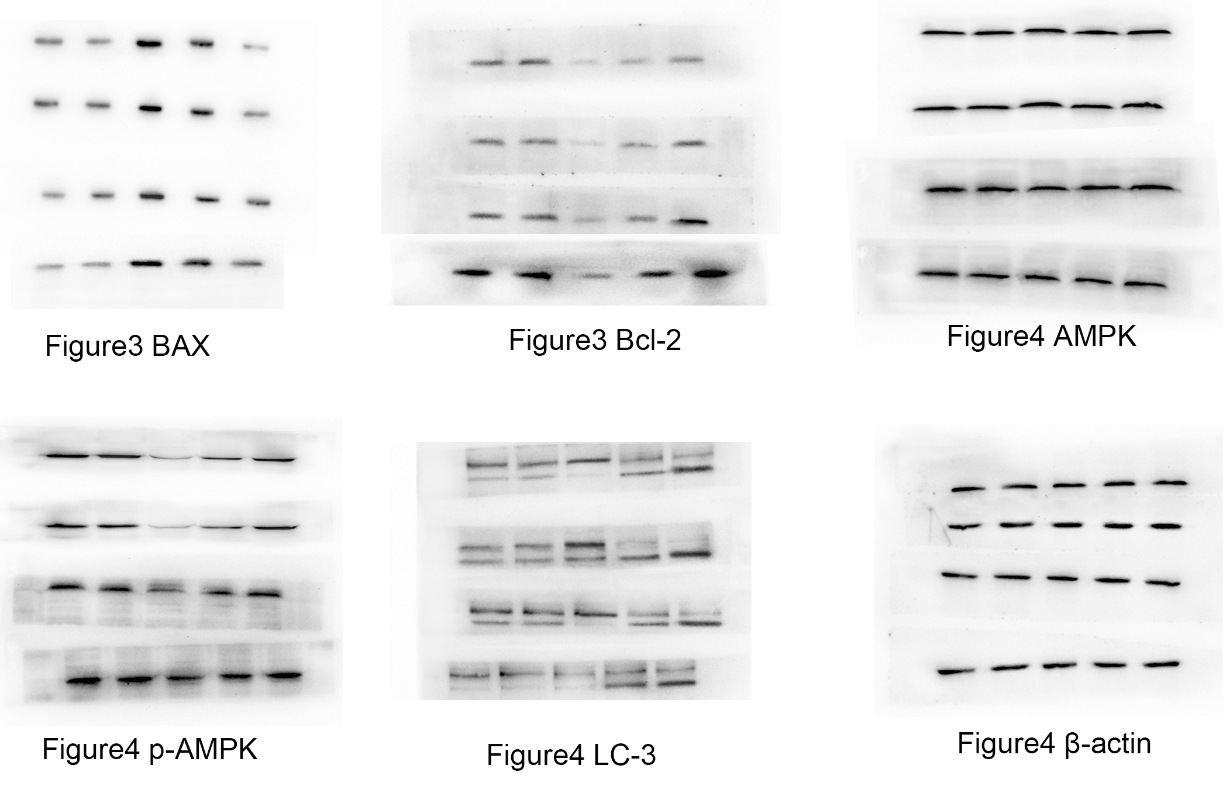

Supplement: Supplementary Materials — The uncropped bolts of all protein blots in Figures. [file 7467156.f1.docx]
